# Photoelectrocatalysis for Hydrogen Evolution Ventures into the World of Organic Synthesis

**DOI:** 10.1002/gch2.202400012

**Published:** 2024-04-14

**Authors:** Giuseppe Sportelli, Miriam Marchi, Paolo Fornasiero, Giacomo Filippini, Federico Franco, Michele Melchionna

**Affiliations:** ^1^ Department of Chemical and Pharmaceutical Sciences University of Trieste via Licio Giorgieri 1 Trieste 34127 Italy; ^2^ Department of Science, Technology and Society University School for Advanced Studies IUSS Pavia Piazza della Vittoria 15 Pavia 27100 Italy; ^3^ Center for Energy Environment and Transport “Giacomo Ciamician” and ICCOM‐CNR Trieste Research Unit University of Trieste via Licio Giorgieri 1 Trieste 34127 Italy

**Keywords:** hydrogen, hydrogenations, organic syntheses, photoelectrochemistry

## Abstract

The use of light as a catalytic prompt for the synthesis of industrial relevant compounds is widely explored in the past years, with a special consideration over the hydrogen evolution reaction (HER). However, semiconductors for heterogeneous photocatalysis suffer from fast charge recombination and, consequently, low solar‐to‐hydrogen efficiency. These drawbacks can be mitigated by coupling photocatalysts with an external circuit that can physically separate the photogenerated charge carriers (electrons and holes). For this reason, photoelectrochemical (PEC) production of hydrogen is under the spotlight as promising green and sustainable technique and widely investigated in numerous publications. However, considering that a significant fraction of the hydrogen produced is used for reduction processes, the development of PEC devices for direct in situ hydrogenation can address the challenges associated with hydrogen storage and distribution. This Perspective aims at highlighting the fundamental aspects of HER from PEC systems, and how these can be harnessed toward the implementation of suitable settings for the hydrogenation of organic compounds of industrial value.

## Graphical abstract

Photoelectrochemistry (PEC) is under the spotlight as promising green and sustainable technique for the production of hydrogen. However, issues related to its storage and distribution promote the development of strategies that may couple also direct H_2_ utilization. This Perspective showcases the recent advancements concerning the in situ hydrogenation of organic molecules though PEC devices.

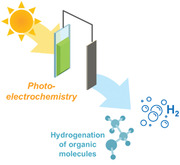

## 1 Introduction

Due to its extensive demand on global scale, hydrogen (H_2_) plays a pivotal role in societal development and growth. According to the International Energy Agency (IEA), the total hydrogen consumption reached 95 Mt in 2022, mainly exploited as chemical feedstock for ammonia (also for related fertilizers production) and methanol synthesis as well as reducing agent for iron processing.^[^ ^1^ ^]^ However, the hydrogen demand is still covered mostly by the strongly CO/CO_2_‐emissive steam methane reforming.^[^ ^1^, ^2^, ^3^, ^4^ ^]^ More sustainable alternative strategies for the production of H_2_, such as electrolysis and biomass reforming, are under development to fulfil decarbonization targets.^[^ ^1^, ^2^, ^3^, ^4^ ^]^ In parallel, the environmental and economic impact of these processes can be further reduced by using renewable electricity and/or light to trigger the hydrogen evolution reaction (HER).^[^ ^3^, ^5^, ^6^ ^]^ It is recognized that versatile and environmental‐friendly production strategies might pave the way for the use of H_2_ as clean energy carrier, whose potential is not fully exploited yet, because of complexity in efficient storage and transportation and lack of market competitiveness with the conventional thermal processes based on fossil fuels.^[^ ^1^, ^6^ ^]^

The development of a new ‘hydrogen economy’ is also hampered by safety issues mainly related to hydrogen storage in its natural form (pressurized tanks), due to the high flammability combined with the risk of explosion.^[^ ^7^ ^]^ Liquid hydrogen carriers^[^ ^4^, ^8^, ^9^, ^10^ ^]^ and solid‐state hydrogen storage^[^ ^4^, ^11^ ^]^ could represent two viable solutions. On the other hand, an attractive new approach consists in the development of strategies based on an in situ production and utilization of H_2_, which are particularly suitable for small scale applications, such as those of fine chemicals synthesis. Photoelectrocatalysis embraces many concepts that address possible solutions to the above‐mentioned issues. In fact, implementation of large‐size (photo)eletrolyzers brings on many additional technical challenges, linked not only to the chemistry but also to the electrochemical aspects. From an industry point of view, the scaling up of laboratory‐based protocols implies a significant economic effort, in particular for the ones based on sophisticated scientific approaches. Considering all this, short‐term translation of photoelectrocatalytic hydrogenation to industrial level seems more viable for fine chemicals production where production demand is less than other sectors, and the economic benefits can be maximized already relying on medium‐size photoelectrochemical setups.

It is therefore not surprising that it currently occupies a central position for the green energy technology development, including light‐to‐hydrogen conversion devices. Indeed, the almost‐even worldwide distribution of the solar irradiance entails the possibility of building decentralized infrastructures that can produce H_2_ at will, eventually exploiting it directly in synthetic processes.^[^ ^4^ ^]^

**Figure 1:**
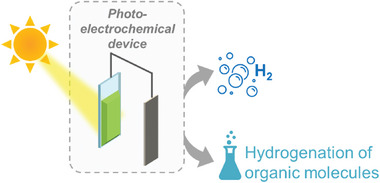
Schematic representation of the solar‐driven electrocatalytic H_2_ evolution and hydrogenation of organic compounds.

This Perspective aims at highlighting new strategies for photoelectrochemical systems to combine the production of hydrogen with in situ hydrogenation of organic substrates. This concept somehow mimics recent and advanced approaches used in photocatalysis for simultaneous generation of H_2_ and production of added value products (**Figure** **1**).^[^ ^12^, ^13^, ^14^ ^]^

## 2 Photoelectrochemical Water Splitting

In contrast to the energy‐intensive production methods based on fossil resources, H_2_ generation from electricity‐driven water splitting is considered a promising technology to produce H_2_ with minimum energy inputs. Despite the inherent advantages, this process is still largely of academic interest, while the industrial use of electrochemical water splitting accounts for only 4% of the world's total hydrogen production.^[^ ^1^ ^]^ Reasons for such polarized interest is manifold as described below.

The overall water splitting consists of two half‐cell reactions: the hydrogen evolution reaction (HER) occurs at the cathode and the oxygen evolution reaction (OER) at the anode. The latter represents the actual thermodynamic challenge, as its standard potential (+1.23 V) corresponds to an energy of 237 kJ mol^−1^, which makes up for the total energy barrier to the process (by definition, the proton reduction under standard conditions sits at 0 V potential). However, under operative conditions the process requires an energy larger than the theoretical 1.23 V, which relates to the kinetic constraints and that depend on several parameters, including the catalytic material used, making the design of the catalysts one of the most critical aspects.^[^ ^15^ ^]^

**Figure 2:**
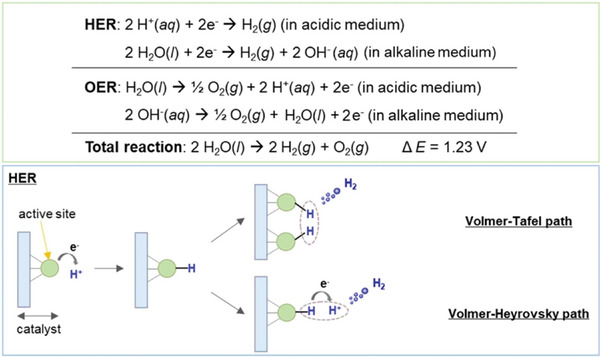
Top panel: electrochemical water splitting reactions in acidic and alkaline conditions. Bottom panel: schematic representation of mechanistic pathways of HER in acidic medium. Adapted under terms of the CC BY 4.0 license.^[^ ^16^ ^]^ Copyright 2022, The Authors.

The HER and OER half reactions are shown in **Figure** **2** for both acidic and alkaline environment.^[^ ^16^ ^]^ Importantly, the proposed HER mechanism in acidic media consists in an initial H^*^ (in which the asterisk indicates a bound H atom) adsorption on the catalyst surface (Volmer step) followed by two competitive pathways: *i*) H^*^ combining with a proton and an electron into H_2_ (Heyrovsky step); *ii*) two adsorbed H^*^ can also bond into H_2_ (Tafel step). Unlike acidic conditions, HER mechanism in an alkaline medium is hindered in the first step due to slow water dissociation.^[^ ^16^, ^17^, ^18^, ^19^ ^]^

At a first sight, production of H_2_ through water splitting shines as a truly carbon neutral process, but, in practice, CO_2_ emission may occur upstream, as the required electrochemical power for water electrolysis is in most cases obtained from non‐renewable sources. A significant decrease in the environmental impact thus hinges on the use of renewable energy sources for water electrolysis, such as solar energy. Solar energy may be used to trigger a direct ‘pure’ photocatalytic water splitting, without relying on electrochemical cell devices, although in this case additional challenges arise.^[^ ^20^ ^]^ Photoelectrochemical (PEC) systems emerge as a modern method for solar H_2_ generation, synergistically exploiting advantages of both photo‐ and electrocatalysis.

A PEC device relies on the use of semiconductors as photoelectrodes, and it requires two distinct electrodes (an anode for the oxidation and a cathode for the reduction) separated by a membrane to divide the production of H_2_ and O_2_.^[^ ^21^ ^]^ Semiconductor materials with suitable bandgaps can serve as both electrocatalysts for water splitting and light absorbers, capable of harvesting photons with energy greater than their bandgap energy. Thus, the main steps of a PEC system include: *i*) light absorption by the photoelectrode; *ii*) separation and migration of charge carriers; *iii*) surface reactions and desorption of H_2_ and O_2_.^[^ ^22^ ^]^

**Figure 3:**
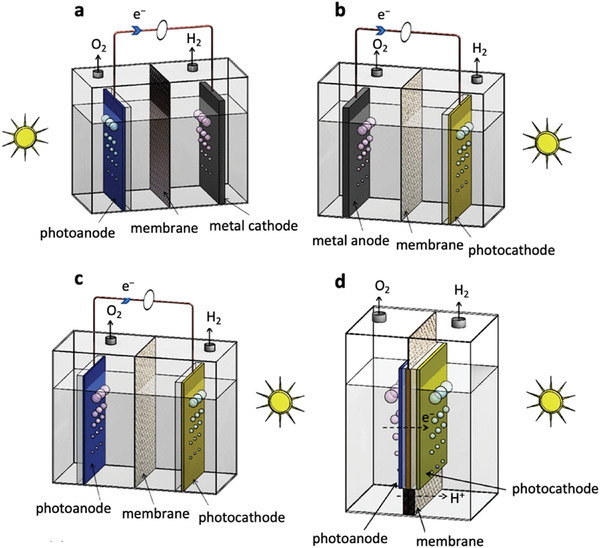
Schematic illustration of some PEC devices: a) photoanode with metal cathode, b) photocathode with metal anode, c) wired tandem PEC cell with photoanode and photocathode, and d) wireless tandem PEC device. Adapted with permission.^[^ ^21^ ^]^ Copyright 2018, Elsevier Ltd.

Typical PEC systems may consist of a single photoelectrode (photocathode or photoanode) connected to a counter electrode, or a tandem design featuring both a photoanode and a photocathode, designed to improve the photopotential and light absorption (see **Figure** **3**).^[^ ^21^, ^23^, ^24^ ^]^ A more detailed discussion of PEC water splitting devices will not be provided herein, as it falls outside the scope of this Perspective.

Significant efforts are currently underway to achieve high STH efficiencies combined with long‐term operating stability. The highest STH efficiency (19.3%) for unbiased water splitting was reported employing multijunction monolithic PEC device with III–V semiconductor.^[^ ^25^ ^]^ Despite such significant milestone, the considerable cost associated to III–V semiconductor materials hinders their commercialization. Conversely, binary metal oxides (e.g., Fe_2_O_3_, WO_3_, Cu_2_O), chalcogenides (e.g., CdS, MoS_2_, WSe_2_), oxynitrides (e.g., TaON, LaTiO_2_N), multinary compounds (e.g., BiVO_4_, Fe_2_TiO_5_, NiMoO_4_) and carbon‐based materials have been extensively investigated as low‐cost, effective and robust catalysts.^[^ ^16^, ^26^, ^27^, ^28^, ^29^, ^30^ ^]^

The main setbacks to address are related to both the system configurations and the material features. As far as the latter is concerned, typical issues are the limited light absorption, the high recombination rates of charge carriers, the slow charge transfer at the semiconductor–liquid interface, the low photocurrent densities, the limited efficiencies, the poor stability and the photocorrosion. All these aspects are serious hurdles for the development of effective devices.^[^ ^4^, ^16^ ^]^ In this context, the choice of the materials is one game‐changer, addressing not only technical constrains, but also geopolitical availability and oscillating market cost. Strategies to boost activity and stability are based on tailoring the semiconductor's structure, by means of doping, surface modification with defects or functional groups, nanostructuring and heterojunction formation.^[^ ^27^, ^31^, ^32^ ^]^ For instance, the doping of BiVO_4_ with phosphate anions has proved to improve the charge transfer and thus increase the photocurrent density by ≈30 times compared to the pristine BiVO_4_.^[^ ^33^ ^]^ Similarly, superior activity for water splitting was achieved through photoelectrode heterostructuring, such as by decorating *p*‐Si microwires with CoSe_2_ nanorods^[^ ^34^ ^]^ or by coupling WO_3_ and BiVO_4_.^[^ ^35^, ^36^ ^]^ To sum up, it has become evident that the design of optimal nanoscale catalysts represents a powerful tool with the potential to promote ‘exact’ chemical manufacturing with minimal waste, thereby tuning the electronic, optical and catalytic features of the materials.^[^ ^15^, ^27^, ^37^, ^38^ ^]^

## 3 Photoelectrochemical Hydrogenation of Organic Compounds

While recent advancements in PEC HER technologies endorse optimistic prospects for more sustainable energy solutions, it is important to acknowledge that storing gaseous H_2_ remains a considerable challenge due to its inherently elusive nature and the demanding requirements for containment and safety measures.^[^ ^4^ ^]^

**Figure 4:**
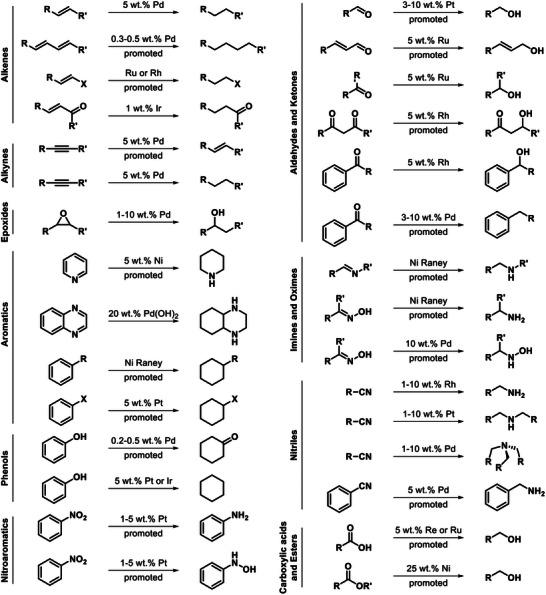
Hydrogenation protocols traditionally applied at industrial level. Adapted with permission.^[^ ^40^ ^]^ Copyright 2016, Wiley‐VCH GmbH.

An appealing yet underexplored solution to this issue contemplates the direct consumption of the electrogenerated H_2_ or of the metal‐hydride intermediate obtained from water reduction for in situ synthetic purposes. As we mentioned before, a large fraction of the global H_2_ demand is actually intended to cover hydrogenation reactions, including fine chemical industry (see **Figure** **4**) together with the well‐known ammonia and methanol syntheses.^[^ ^1^, ^39^ ^]^ Nevertheless, conventional thermocatalytic activation of H_2_ still relies on harsh (e.g., high pressures and temperatures) and energy‐intensive protocols and frequently requires precious metals and promoters to achieve adequate activity and selectivity (Figure 4).^[^ ^39^, ^40^ ^]^

**Figure 5:**
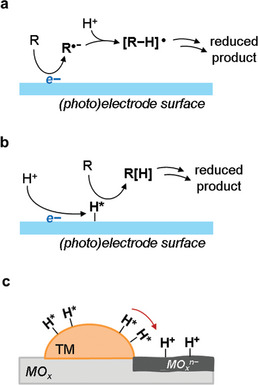
Main reduction pathways for (photo)electrocatalysis are shown a,b) and spillover effect for H^*^ intermediates c). TM = transition metal; MO*_x_* = reducible metal oxide support; MO*_x_^n−^* = reduced superficial sites; R = organic molecule.

Photoelectrocatalysis can offer a milder alternative to the traditional thermal pathways, combining photoabsorber materials with electrochemical reactions to achieve enhanced and efficient catalytic transformations.^[^ ^41^, ^42^, ^43^, ^44^ ^]^ In the case of (photo)electrochemical reductions, indeed, the electrons accumulated on the electrodes’ surface can be directly transferred to a water‐soluble organic substrate, yielding a radical anion intermediate, followed by a protonation step in which water acts as proton donor (**Figure** **5**a).^[^ ^45^ ^]^ Alternatively, the reaction mechanism can proceed through an adsorbed H^*^ intermediate on the electrode surface *via* Volmer step, as it occurs for HER (Figure 5b).^[^ ^46^, ^47^, ^48^, ^49^ ^]^ Specifically, such hydride intermediates can couple with the reducible moiety in the organic substrate, resulting in hydrogenation. It is worth remarking that these ‘electron‐rich’ H^*^ species can also freely migrate over the metal's surface and, in presence of reducible supports, activate the hydrogen ‘spillover’ process (see Figure 5c).^[^ ^39^, ^50^, ^51^ ^]^ Such phenomenon strongly contributes to improve the intermediates’ lifetime, ensuring higher conversion rates.^[^ ^50^, ^52^ ^]^

Only few reports of photoelectrochemical hydrogenations (PECH) involving H^*^ intermediates are currently available in literature, hinting that such a subject provides a lot of room for breakthroughs.

**Scheme 1:**
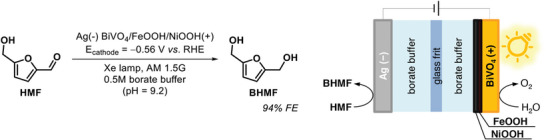
PECH of 5‐hydroxymethylfurfural (HMF) to 2,5‐bis(hydroxymethyl)furan (BHMF) with graphical description of the corresponding PEC system. RHE = reversible hydrogen electrode.

One of the milestones was in fact proposed only in 2016, using a divided PEC cell with a *n*‐type BiVO_4_/FeOOH/NiOOH photoanode and a high‐surface‐area Ag cathode (**Scheme** **1**), able to trigger the hydrogenation of 5‐hydroxymethylfurfural (HMF) to 2,5‐bis(hydroxymethyl)furan (BHMF),^[^ ^53^ ^]^ which is a very useful substrate for biodiesels formulation as well as for polymerizations.^[^ ^48^, ^54^, ^55^ ^]^ Notably, the PECH proceeds with Faradaic efficiency (FE) of 94% and selectivity of 95%,^[^ ^53^ ^]^ even accounting the possible competitive oxidation to 2,5‐furandicarboxylic acid observed with Pt as counter‐electrode (in spite of Ag).^[^ ^56^ ^]^

**Scheme 2:**
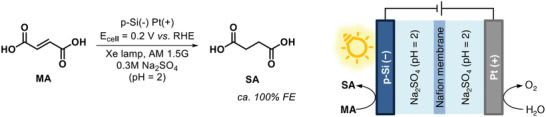
PECH of maleic acid (MA) to succinic acid (SA) with graphical description of the corresponding PEC system. RHE = reversible hydrogen electrode.

The reduction of C═C bonds through PEC approaches has been attracting increasing attention, considering that this process is of paramount importance also for commodity industry.^[^ ^57^ ^]^ On this regard, Chen et al. successfully explored the use of *p*‐Si nanowire array photocathode for the PECH of maleic acid (MA) to succinic acid (SA) with FE of ≈100% (**Scheme** **2**).^[^ ^57^ ^]^

**Scheme 3:**
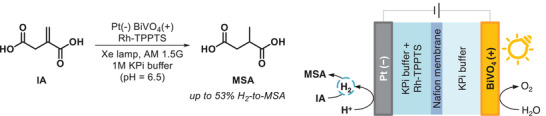
Reduction of itaconic acid (IA) to methyl succinic acid (MSA) with graphical description of the corresponding PEC system, including the formation of H_2_ on the anode surface that serves as reducing agent via Rh‐TPPTS. Rh‐TPPTS = rhodium trisodium 3,3′,3′′‐phosphanetriyltri(benzene‐1‐sulfonate); KPi = potassium phosphate.

A more in‐depth investigation over this class of transformations has been recently offered by Abdi and colleagues.^[^ ^58^, ^59^ ^]^ In particular, the design of an energy‐efficient reduction of itaconic acid (IA) to methyl succinic acid (MSA) was successfully applied. The system consisted of a tailored photoelectrocatalytic HER coupled with a homogenous rhodium trisodium 3,3′,3′′‐phosphanetriyltri(benzene‐1‐sulfonate) (Rh‐TPPTS) complex (**Scheme** **3**).^[^ ^58^, ^59^ ^]^ The device was first evaluated on the basis of lifecycle assessment of net energy parameters entangled in the fabrication process^[^ ^58^ ^]^ and, after definition of the best components from the techno‐economical point of view, the assembly was tested for the reaction.^[^ ^59^ ^]^ An impressive constant production over prolonged times of ≈50 µmol of MSA (53% H_2_‐to‐MSA conversion) was achieved by using BiVO_4_ photoanodes with a 1.26 V bias (Scheme 3).^[^ ^59^ ^]^

Recently, PEC strategies have been combined also with biocatalysis, where a first step consisting in the reduction of biological co‐factors (mainly flavin mononucleotide, FMN, and 1,4‐dihydronicotinamide adenine dinucleotide, NADH) occurred at the electrodes’ surface. As these ‘vectors’ travel in the aqueous electrolyte, they can interact with enzymes, promoting the hydrogenation reaction on organic substrates.^[^ ^60^, ^61^, ^62^ ^]^ The photoelectrocatalytic method was also applicable to the enantioselective hydrogenation of C═C bonds via ene reductase, taking place at the photocathodic side of a bias‐free PEC with a silicon photovoltaic/indium tin oxide (*p*‐Si/ITO) electrode, while lignin refinery occurs on the α‐Fe_2_O_3_ photoanode.^[^ ^61^ ^]^ As an interesting alternative, plastic waste could function as sacrificial electron donor.^[^ ^62^ ^]^

Despite these promising results, the application of photoelectrocatalytic hydrogenations at an industrial scale is still limited by the following issues: *a*) the lack of efficient photoelectrodes with high durability and stability; *b*) the competition with HER reduces the conversion efficiency of the process; *c*) the presence of several reaction pathways leads to multiple products, lowering the selectivity and increasing separation costs; *d*) the necessity of an aqueous electrolyte for charge transport phenomena can pose a solubility limitation, narrowing the choice of organic substrate/target molecules; *e*) the need for precisely designed cells that do avoid undesired reactions at the counter‐electrode; *f*) the reaction rates of PECH are generally too slow to satisfy large‐scale industrial production.^[^ ^41^, ^63^ ^]^

Innovative strategies for rational design of the catalytic system are required to tackle these challenges. According to recent findings achieved in related fields, such as CO_2_ electroreduction, the protection of the photocathode surface with highly stable hydrophobic molecular films may represent a promising strategy to improve the stability of the photoelectrode (point *a*) and limit HER in aqueous media (point *b*).^[^ ^64^ ^]^ In parallel, careful design of the catalytic material itself must be approached with the utmost care to improve the selectivity toward a specific product (point *c*). Besides optimizing light absorption on the photoabsorbing side (with strong preference for the solar spectrum), the hydrogenation catalyst has to be adequately tailored to improve H^*^ stabilization. While a metal component mainly functions as mediator for transfer of hydrogen atoms via H spillover phenomena, the superficial nature of these intermediates plays a pivotal role in hydrogenation selectivity.^[^ ^52^, ^65^ ^]^ For example, the density of defects (oxygen vacancies) on the CeO_2_, and consequently the Ce^3+^/Ce^4+^ ratio, as well as the specific metal–metal oxide interface are proven to be tightly responsible for hydrogenation efficiency.^[^ ^52^, ^66^ ^]^ Such phenomenon has recently been used to hydrogenate CO_2_ by adopting a dual‐active site strategy, where CeO_2_ served to adsorb CO_2_, and could reduce it to CO via a mechanism based on the spillover of H from Pt nanoclusters.^[^ ^67^ ^]^

In addition to the catalytic system, further progress in the engineering design of the PEC reactor needs to be improved in order to overcome practical issues related to the chemical compatibility of the organic substrates with the reaction medium as well as the slow PECH rates (points *d–f*). The great potentiality has been already demonstrated through the above examples, and encouragingly in similar electrocatalytic contexts.^[^ ^45^, ^68^, ^69^, ^70^ ^]^

## 4 Outlook and Conclusions

As global H_2_ demand continues to rise year after year, scientists should relentlessly seek new technologies that make possible to fit the market trends while also addressing environmental and sustainability issues.

The combined use of light and electricity (with special consideration for renewable energy sources) in a photoelectrocatalytic setting seems to be a promising strategy to efficiently perform challenging processes with high H_2_ production efficiency. While important *per se*, the competence of photoelectrocatalytic materials toward H_2_ formation can also be deployed to address the in situ hydrogenation of organic molecules or units that are found in industrial relevant products. Such a strategy hinges on tailored catalyst structure, and it calls for material design that combine several components.

The above‐mentioned examples are the first milestones in this field, which will be of value to examine all the criticalities that may arise during projecting of suitable PEC systems for organic synthesis. Nevertheless, the limited number of PEC‐related publications suggest that the subject is on its infancy state, opening the door to countless opportunities yet to be explored.

Apart from inherent activity of the photoelectrocatalytic material, improvements of stability and efficiency in terms of PEC design and electrode fabrication are essential elements to consider in order to fit industrial requirements. Moreover, a precise selection of composition, electronic and structural features of electrodic materials might finally end up in smart devices that can switch from H_2_ evolution to hydrogenations on command.

## Conflict of Interest

The authors declare no conflict of interest.
